# Establishing a Demographic, Development and Environmental Geospatial Surveillance Platform in India: Planning and Implementation

**DOI:** 10.2196/publichealth.9749

**Published:** 2018-10-05

**Authors:** Shikha Dixit, Narendra K Arora, Atiqur Rahman, Natasha J Howard, Rakesh K Singh, Mayur Vaswani, Manoja K Das, Faruqueuddin Ahmed, Prashant Mathur, Nikhil Tandon, Rajib Dasgupta, Sanjay Chaturvedi, Jaishri Jethwaney, Suresh Dalpath, Rajendra Prashad, Rakesh Kumar, Rakesh Gupta, Laurette Dube, Mark Daniel

**Affiliations:** 1 Research SOMAARTH Demographic, Development and Environmental Surveillance Site The INCLEN Trust International New Delhi India; 2 Research Epidemiology The INCLEN Trust International New Delhi India; 3 Department of Geography Faculty of Natural Sciences Jamia Millia Islamia New Delhi India; 4 Sansom Institute for Health Research Division of Health Sciences University of South Australia Adelaide Australia; 5 South Australian Health and Medical Research Institute Adelaide Australia; 6 Academics Department Aditya Diagnostic & Hospital Dibrugarh India; 7 National Cancer Registry Program National Centre for Disease Informatics and Research Indian Council of Medical Research Bangalore India; 8 Department of Endocrinology All India Institute of Medical Sciences New Delhi India; 9 Centre of Social Medicine and Community Health Jawaharlal Nehru University New Delhi India; 10 Department of Community Medicine University College of Medical Sciences University of Delhi New Delhi India; 11 Department of Research Indian Council for Social Science Research New Delhi India; 12 Department of Health Panchkula India; 13 Office of Chief Medical Officer Department of Health Palwal India; 14 Indian Council of Medical Research New Delhi India; 15 Office of Chief Minister Chandigarh India; 16 McGill Center for the Convergence of Health and Economics McGill University Montreal, QC Canada; 17 Centre for Research and Action in Public Health Health Research Institute University of Canberra Canberra Australia; 18 Department of Medicine St. Vincent’s Hospital The University of Melbourne Melbourne Australia

**Keywords:** geospatial surveillance, health and nonhealth data harmonization, spatial epidemiology, participatory GIS, caste, socioeconomic transition, ground truthing, built environment

## Abstract

**Background:**

Inadequate administrative health data, suboptimal public health infrastructure, rapid and unplanned urbanization, environmental degradation, and poor penetration of information technology make the tracking of health and well-being of populations and their social determinants in the developing countries challenging. Technology-integrated comprehensive surveillance platforms have the potential to overcome these gaps.

**Objective:**

This paper provides methodological insights into establishing a geographic information system (GIS)-integrated, comprehensive surveillance platform in rural North India, a resource-constrained setting.

**Methods:**

The International Clinical Epidemiology Network Trust International established a comprehensive SOMAARTH Demographic, Development, and Environmental Surveillance Site (DDESS) in rural Palwal, a district in Haryana, North India. The surveillance platform evolved by adopting four major steps: (1) site preparation, (2) data construction, (3) data quality assurance, and (4) data update and maintenance system. Arc GIS 10.3 and QGIS 2.14 software were employed for geospatial data construction. Surveillance data architecture was built upon the geospatial land parcel datasets. Dedicated software (SOMAARTH-1) was developed for handling high volume of longitudinal datasets. The built infrastructure data pertaining to land use, water bodies, roads, railways, community trails, landmarks, water, sanitation and food environment, weather and air quality, and demographic characteristics were constructed in a relational manner.

**Results:**

The comprehensive surveillance platform encompassed a population of 0.2 million individuals residing in 51 villages over a land mass of 251.7 sq km having 32,662 households and 19,260 nonresidential features (cattle shed, shops, health, education, banking, religious institutions, etc). All land parcels were assigned georeferenced location identification numbers to enable space and time monitoring. Subdivision of villages into sectors helped identify socially homogenous community clusters (418/676, 61.8%, sectors). Water and hygiene parameters of the whole area were mapped on the GIS platform and quantified. Risk of physical exposure to harmful environment (poor water and sanitation indicators) was significantly associated with the caste of individual household (*P*=.001), and the path was mediated through the socioeconomic status and density of waste spots (liquid and solid) of the sector in which these households were located. Ground-truthing for ascertaining the land parcel level accuracies, community involvement in mapping exercise, and identification of small habitations not recorded in the administrative data were key learnings.

**Conclusions:**

The SOMAARTH DDESS experience allowed us to document and explore dynamic relationships, associations, and pathways across multiple levels of the system (ie, individual, household, neighborhood, and village) through a geospatial interface. This could be used for characterization and monitoring of a wide range of proximal and distal determinants of health.

## Introduction

### Background

Inadequate administrative health data, suboptimal public health infrastructure, rapid and unplanned urbanization, environmental degradation, and poor penetration of information technology make the tracking of health and well-being of populations in the developing countries challenging [1-4]. Health surveillance capacities remain one of the major barriers in collating contextual evidences for identifying the pathways of health problems and assessing the true magnitude of the socioeconomic impact of diseases; new technologies and innovations hold promise for finding solutions in such environments [1,2,5,6]. Surveillance of behavioral, socioeconomic, and environmental determinants of health is further limited in terms of capacity to develop infrastructure and collect and interpret the information in resource-constrained settings [1,6,7].

The US Center for Disease Control and Prevention has recently advocated for the establishment of comprehensive surveillance architectures for emerging infectious diseases and chronic conditions, particularly those associated with lifestyle, incorporating wider (distal and proximal) determinants of health and well-being [2]. Integrative surveillance of diverse environmental factors with a “whole-of-society” convergence framework is likely to be informative of the factors that contribute to occurrence, sustenance, and progression of communicable and noncommunicable diseases [8].

A geographic information system (GIS) enables an integrated comprehensive surveillance platform that allows rapid integration of data from disparate sectors and sources with the potential to contribute to improving the understanding of diverse disease exposures [9-16]. Although geospatial technologies have been explored and experimented with in several studies conducted in developed countries [12], there is limited experience from the developing countries due to reasons like lack of georeferenced administrative health datasets and postal codes, unavailability of trained technical manpower, and the complex morphologies of human habitations, particularly rural settings [17-21].

Between 2009 and 2015, the International Clinical Epidemiology Network (INCLEN) Trust International established a comprehensive SOMAARTH Demographic, Development, and Environmental Surveillance Site (DDESS) in a rural North Indian setting (District Palwal, Haryana). As a surveillance platform, SOMAARTH (the word SOMAARTH is a Sanskrit word meaning synergy between economic development and health) DDESS aims to allow monitoring and interpretations of synergetic and complex relationships between the environment, society, regional development, economics, and health status of the population over time (Multimedia Appendix 1).

**Figure 1:**
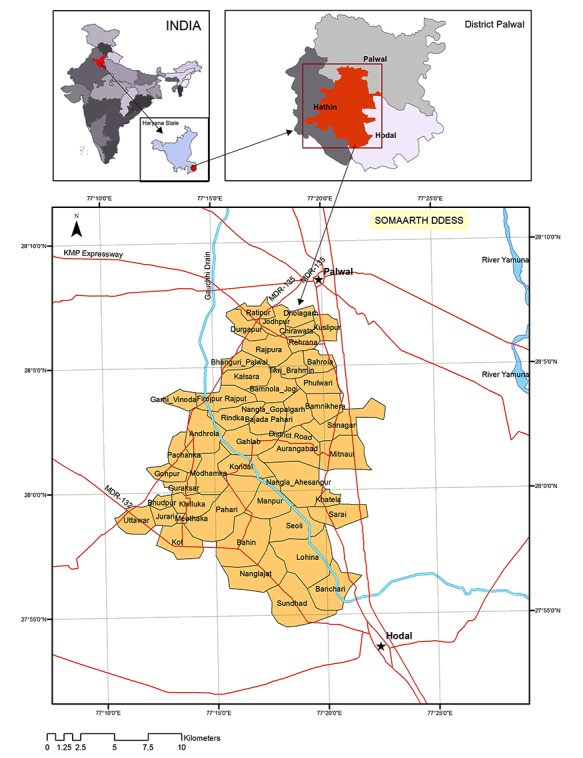
Location of the SOMAARTH Demographic, Development, and Environmental Surveillance Site (DDESS).

Building on the existing global experiences, this paper describes the feasibility of establishing a GIS-integrated surveillance platform, SOMAARTH DDESS, and shares the learnings gained in the context of a resource-constrained rural North Indian setting.

### Surveillance Site Location, Coverage, and Characteristics

The SOMAARTH DDESS (District Palwal, Haryana, India) is located about 80 km from the Delhi border on the Delhi-Agra National Highway 2 (NH-2). This site is located between 27°53′59.46″N to 28°7′30.02″N latitude and 77°10′2.95″E to 77°22′47.35″E longitude spanning over 251.7 sq km of area (Figure 1) and includes 51 villages from 3 administrative blocks (Hodal, Hathin, and Palwal) of the district. As per the 2011 census, the decadal population growth rate of Palwal district was 25.7% as against the Indian national average of 17.7%; over three-quarters (77.3%) of the district population is rural [22]. The climate of the study area can be classified as arid steppe hot according to Köoppen-Geiger Classification system [23]. The Western Peripheral Expressway (Kundli-Manesar- Palwal Expressway) traverses through the northern tip of the study site, and the proposed Special Economic Zones along the expressway are projected to boost local industrial and business growth [24].

## Methods

### Tools and Techniques

Google Earth open source imagery and Survey of India Palwal district map of 1:50,000 scale were utilized for preparing the initial maps. QuickBird very high-resolution (<1 m), multispectral, radiometrically corrected, and projected satellite imagery for the period March-May 2012 was procured from the National Remote Sensing Centre, India.

Environmental Systems Research Institute Arc Map Version 10.3 (ESRI, Redland, CA, USA) [25] and QGIS Version 2.1 (QGIS Development Team) [26] software were used for GIS analysis. MetOne E-sampler 9800 for ambient particulate matter (PM_2.5_) and meteorological data (wind speed and direction, temperature, and relative humidity), UCB-PATS+ for household PM_2.5_, MAXIM i-buttons for stove usage monitoring, and DJI Phantom-1 for recording particle dispersion and temperature inversions were utilized for establishing an air quality monitoring system.

### Surveillance Architecture

SOMAARTH surveillance platform architecture was established for tracking the distal and proximal determinants of health through 3 key surveillance activities (Multimedia Appendix 2): (1) development and built environmental surveillance that encompasses land use, including commercial, industrial, institutional, educational, transportational, and contextual structures; (2) demographic and health surveillance, including size, structure, distribution, and population health; and (3) physical environmental factors including the indoor and outdoor air quality, ambient metrological data (ie, temperature, humidity, and wind direction), and water and sanitation.

A three-tier surveillance architecture was conceived using geospatial interfacing to enable incorporation of domain-specific areas, including the following layers: data collection (input layer), data management (application layer), and data harmonization (database layer). Datasets were prepared to permit relational documentation across each layer and to dynamically integrate additional information from research projects, health facilities, and institutional records in a timely manner as datasets were made available to the research team.

### Data System Development Steps

A multidisciplinary expert group, the Central Coordination Team, was formed; it comprised specialists from the fields of public health, epidemiology, pediatrics, geospatial science, human geography, anthropology, environmental science, urban planning, management, and social sciences. The Central Coordination Team guided the establishment processes and the development of a conceptual framework. For surveillance site selection, a rural area circumscribed by the three major roads and having potential for rapid economic development was identified. Official permission from the state government and district administration was gained prior to undertaking field activities. Prior approval from the competent state or national authorities and from the community leaders is mandatory for setting up the demographic surveillance sites [27]. The progress of the SOMAARTH surveillance platform consisted of four major steps: (1) site preparation, (2) data construction, (3) data quality assurance, and (4) data update and maintenance system.

#### Step 1: Site Preparation (18 months, October 2009-March 2011)

Developing the surveillance platform was a long-term commitment and required continuous support from the local stakeholders including community members. Initial contact with the village community and administration was established (October 2009), and a partnership was forged over a period of 18 months with the local community leaders through village-level community meetings. Stakeholder engagement established the networks required to later undertake participatory mapping and census processes within the villages.

Three field teams were constituted: Census, GIS, and Environment teams. Teams comprised lead personnel with public health (n=5), geography (n=2), and environmental science (n=1) backgrounds; for field staff, local residents with graduate and undergraduate qualifications were hired (Census, 38; GIS, 11; Environment, 3). Project personnel were trained through 3 separate structured 2-week training programs, which included classroom sessions (20% time) along with hands-on fieldwork (80% training time). A village mapping listing manual, census enumeration guide, and GIS mapping guidelines were prepared to ensure consistency in data collection processes across the site. Separate microplans for collecting datasets pertaining to geospatial, demographic, and environmental domains were prepared, and instruments were finalized after a team of 4 investigators (NKA, MV, FA, and RKS) and 6 field staff piloted field activities in 5 villages over 12 working days.

#### Step 2: Data Construction (38 months, March 2011-April 2014)

**Figure 2:**
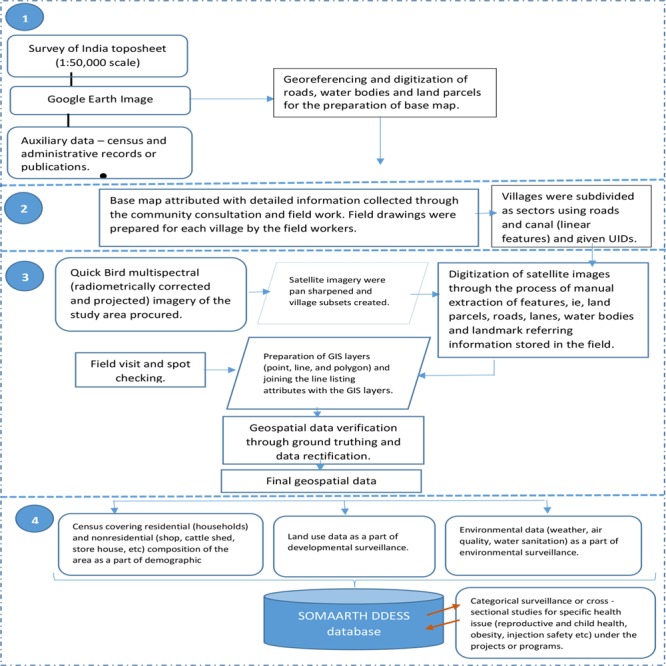
The SOMAARTH Demographic, Development, and Environmental Surveillance Site (DDESS) data system: development and integration of demography, development, and environmental parameters. UID: unique identification number; GIS: geographic information system.

In the absence of administrative datasets, baseline datasets were constructed for establishing a comprehensive surveillance platform (Figure 2).

**Figure 3:**
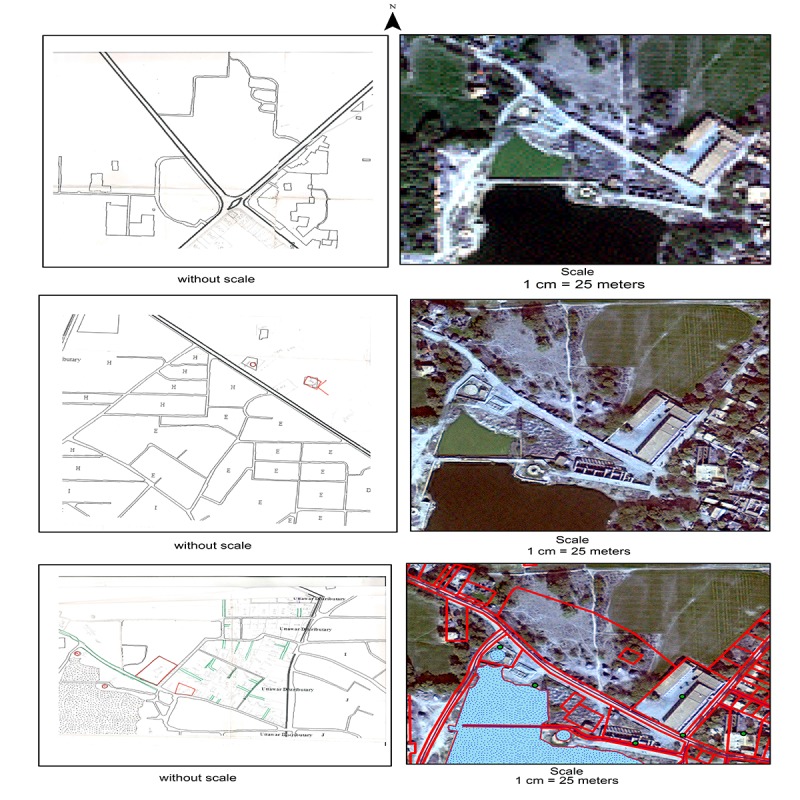
Stepwise development of geospatial datasets at SOMAARTH Demographic, Development, and Environmental Surveillance Site, Palwal, India.

##### Characterization of Rural Environment Through Participatory Mapping and Line Listing (12 Months, March 2011-February 2012)

Participatory mapping and line listing were undertaken simultaneously, covering the residential, nonresidential, vacant, and ruined land parcels. Before starting the data collection processes, base maps [28] were prepared by the GIS Associates utilizing the Survey of India toposheet (scale 1:50,000) and Google Earth imagery depicting locations of major roads and water bodies for all 51 villages. A team of 2 field workers (a mapper and lister) per village collected the information using hard copies of base maps and a line listing tool through community consultations, followed by the field work. Field workers identified the main entry point of the village; oriented themselves as per the directions provided on the hard copy of the base map; and following the left-hand rule, systematically captured roads, lanes, water bodies, and landmarks to prepare a detailed field drawing of the village.

Participatory mapping assisted in the subdivision of villages into sectors, resulting into around 50-500 (population of approximately 100-2000 persons) contiguous land parcels in the core habitation area and taking roads as a boundary of demarcation. However, outer village sectors were sparsely built (0-50) land parcels. Sectors were given unique alphabetic identification codes in a systematic clockwise order. Using the left-hand rule, all the residential, nonresidential, and vacant land parcels within each sector were mapped in the form of polygons of relative sizes and shapes of area as informed by the property owner or respondent (Figure 3). Each land parcel occupied in the residential, nonresidential, or mixed activities was given a unique identification number (UID) based on location by prefixing the sector identity and unique numbers in sequential manner following the left-hand rule. This systematic approach later helped in developing location-based addresses for each household and nonresidential features of the study villages. The line list prepared for each land parcel consisted of the following details: structure type as per construction (mud, cement, brick) and usage (residential or nonresidential or vacant or ruined), ownership, head of household, religion, caste, gender, and age composition of household members. The Field Supervisor conducted 10% random field-based checks stratified according to the task accomplished by the primary field workers, and the lot quality assurance approach was adopted for accepting or rejecting the lot.

In Figure 3, top two and middle left images show stepwise development of a field drawing and comprise a framework map depicting major roads digitized from Google Earth; an outlining of village sectors; and a field drawing depicting roads, water bodies, and land parcels, respectively. Furthermore, middle right and bottom two images show stepwise development of geospatial data and comprise a multispectral Quick bird satellite image (raw); a processed (pan-sharpened) image for digitization; and digitized road, water body, and land parcel layers overlaid on the processed image, respectively.

##### Geospatial Data Construction (26 Months, September 2011-October 2013)

The analog participatory maps (field drawings) having contextual details of the villages and the line listing having compositional details proved helpful in satellite-based digitization processes for constructing digital, georeferenced, spatial datasets. Due to the lack of property delineation and informal settlements [20,21], automated digitization [29] was not possible for our area; therefore, manual digitization was adopted through combining visual interpretation of satellite imagery and participatory maps [30]. QuickBird multispectral satellite imagery was pan sharpened for improving the spectral quality for digitization processes (see Figure 3). Data was projected in the Universal Transverse Mercator coordinate system (Zone 43N). Different features were stored as separate feature classes, that is, sector (polygon), roads (polygons, line), water bodies (polygons), land parcels (polygon), wells (point), canal and drains (polygon), railway line (line), and burial places and landmarks (point). Land parcel features with their location UID and composition data collected during line listing were joined with the GIS layers. Digitization for all 51 villages was done by a team of 3 GIS research associates. The Program Officer (GIS) conducted a random cross-checking of 10% of the land parcels stratified for every sector in the village in order to take corrective steps.

##### Demographic and Health Data Collection (26 Months, March 2012-April 2014)

Hard copies of high-resolution GIS maps and line listing attributes were supplied to the census teams to operationalize the census of residential and nonresidential features using 2 separate tools in a systematic manner. The sectorwise high-resolution GIS maps (<1:200 scale) facilitated in work allocation and monitoring of census operations. Census forms were tagged with their respective location UID marked on the GIS map. Core variables collected for the residential structures were as follows: basic land parcel information, demographic details of the inhabitants, household structure, details of construction materials, socioeconomic status (SES), domestic animals and other assets owned by the household, water availability and usage, toilet facilities, sanitation, and waste management practices. The self-reported health parameters covered were as follows: details of mental and physical disability, behavioral issues, substance abuse (smoking, alcohol, and other substances), health-seeking patterns, and individuals with a chronic disease (an illness lasting for more than last 6 months) in the household. Core variables for nonresidential land parcels were land use typology and waste management besides the structural features and ownership. Regular structured coordination-cum-troubleshooting interaction occurred between GIS and Census teams every week to detect temporal changes and other feedbacks on the maps in a real-time manner; these meetings helped in the regular rectification of both census and GIS data.

##### Physical Environmental Data Collection

###### Weather and Air Quality Data Collection (Ongoing Since May 2011)

Environmental scientists led the establishment for air quality data monitoring, which covered point-based recording of real-time ambient air quality (PM_2.5_) and other meteorological attributes (ie, temperature, humidity, and wind direction) at 2 fixed locations within the site. GIS maps helped in the site selection for establishing a small weather station within the surveillance site. The system for PM_2.5_ air quality monitoring at the ambient level was upgraded to drone-based observations for monitoring the dispersion of particles (PM_2.5_) at different altitudes and measurement of temperature inversions. Personal exposure monitoring was also carried out in selected female subjects (primary cook) from the site villages [31]. Latitude and longitude information was used for integrating weather and air quality data with the geospatial datasets.

###### Drinking Water and Sanitation Mapping (November-December 2015)

Baseline assessment of two critical components of the village environment (ie, drinking water supply and sanitary conditions of the rural communities) was performed. This survey was undertaken by a geographer with the help of a field worker hired from the local community. SOMAARTH GIS data were used for creating base maps for mapping water and sanitation status, drinking water pipe lines, drainage system (drainage channel and their quality), and liquid and solid (litter) waste spots (ie, open litter of large size covering more than 1 m diameter). Locations of water stagnation and spilling areas were also mapped on the hard copies of GIS maps and later updated within the geospatial datasets.

###### Land Use Mapping (March 2012-April 2014)

GIS Associates assigned an adapted system of land use categorization [32] to each land parcel. The resulting land use classification system included 3 levels. Level I representing “Built-Up Land,” “Agricultural Land,” “Water Bodies,” “Waste Land,” and “Vacant Land.” For example, Built-Up (Level I) was further refined to Level II to include the classifications of “Residential,” “Commercial,” “Industrial,” “Institutional,” “Utilities,” “Services,” “Transportation,” and “Agricultural and Others.” Subsequently, Level II categories were further refined into a Level III classification. The attribute table within the GIS village layer included all land parcels that were characterized within the village. There was another project going on in the area: “Foundational Work for a Brain-to-Society Diagnostics for Prevention of Childhood Obesity and its Chronic Diseases Consequences.” As part of this project, GIS mapping of food environment was done in 9 villages to identify exposures influencing the food intake of study subjects (children aged 6-12 years) [33].

#### Step 3: Data Quality Assurance (26 Months, April 2013-December 2015)

The geospatial data so constructed was subsequently reassessed for land parcel position (location, size, and shape) and attribute accuracy through ground-truthing–based verification exercises. Temporal changes that emerged during the course of the data construction were also incorporated in this exercise.

The methodology for verification of geospatial datasets through community-based ground-truthing was formulated through a pilot study conducted at 6 surveillance villages. Villages for pilot study were selected through a stratified random sampling process as per size (large, >1000 land parcels and small, ≤1000 land parcels) and settlement pattern (linear, circular): 2 each from large linear and large circular groups and 1 each from small linear and small circular groups. Within each selected village, 5.1% (455/8901) samples of total land parcels across all land use categories were selected, keeping the minimum sample size of 30 land parcels per village. The pilot exercise resulted in the development of 2 verification tools (land parcel and road assessment tools) and the associated operational manual. These tools were applied across the DDESS for data verification and refinement of the GIS maps. Discrepancies were recorded and highlighted on the hard copy maps. Refinement of land parcel delineation was done by capturing vacant land adjoining the existing structures (buildings). An updated road network was prepared for the entire DDESS and characterized according to a predefined typology (ie, highway, village road, public and private lanes), surface (ie, metalled, unmetalled, semimetalled), and surface quality (ie, good, average, poor).

After the completion of the verification exercise, the census forms were tagged with the updated geospatial UIDs and rechecked manually to ensure that the census form was accurately integrated with the corresponding geospatial data. The geospatial dataset was again verified using onscreen tools, topology functions, and ground-truthing processes before sending it for entry into the SOMAARTH surveillance data management software. A team of 8 field workers under the supervision of the 3 GIS research associates worked in this activity for 16 months, between February 2014 and May 2015.

#### Step 4: Data Update and Maintenance System

Data collected on the hard copy forms were entered in the specially designed SOMAARTH DDESS software (SOMAARTH-1) developed on HTML or cascading style sheet user interface, personal home page programming language with MySQL database management system. Software included modules on registration of land parcels, user management, survey, quality assurance, query building, reporting (including tabular and graphical), cohort, and multiple project management. Recently updated integrated Web- and Android-based data collection capabilities have made SOMAARTH-1 software a robust package for handling data collection, storage, management, and analysis for large volumes of longitudinal datasets. Considering the large surveillance area, volume, and variety of datasets, 3 data update strategies were put in place: (1) real-time update of datasets under individual projects; (2) annual update covering temporal changes in the land parcels and 6 vital demographic events, including migration (immigration, emigration), birth, death, pregnancy registration, changes in marital status, and change in the head of households; and (3) complete data collection wave (census) covering all data components (ie, built environment, demographic, and health) every 3 years. SOMAARTH DDESS was prepared for its first annual update after completion of the baseline round of census in May 2018.

## Results

### Description of Data Constructs

Some of the unique geospatial data constructs available within the SOMAARTH platform were physical environment (land parcel, water bodies), social (road, rail, public places, religious places), and services (child and mother care centers, rural banks, health facilities, educational institutes, cremation grounds or burial places, others; Table 1). There were a total of 47,007 land parcels spread across 51 villages; these were characterized as residential (26,363/47,007, 56.08%), nonresidential (18,118/47,007, 39.54%), and mixed (2528/47,007, 5.38%) land parcels. The number of land parcels varied between 25 and 3279 per village (median 587; mean 922 [SD 857]) depending on the average population size (mean 3916 [SD 3673]) per village (median 2603; range 89-18,249; Multimedia Appendix 3).

Demographic and health datasets of 199,702 persons residing at the SOMAARTH DDESS were nested within the geospatial dataset. Granular datasets on village- and neighborhood-level ambient air quality (PM_2.5_) were available from year 2012 onwards.

Almost all the villages (48/51, 94%) had ponds locally called *johar*. All the villages were accessible through metalled roads, with an average road density of 2.8 km per sq km of surface area. Moreover, 18 villages had public health facilities; however, every village had one or more private providers (n=234), most of whom were informal or nonqualified. The median distance of public health facilities in the villages, where they were available, was 370 m (range 142 m-1282 m) from the center of the village built-up area. All the 231 water bodies within the SOMAARTH DDESS were highly polluted due to the dumping of solid and liquid wastes generated by the local inhabitants.

**Table 1:** The SOMAARTH Demographic, Development, and Environmental Surveillance Site geospatial database: physical, social, and service constructs of the area.

SOMAARTH GIS^a^ constructs, data domain, and details or local names			GIS representation	Villages, n	GIS features, n
**Physical environment**					
	**Land parcel**				
		Residential	Polygon	51	26,363
		Nonresidential	Polygon	51	18,116
		Mixed	Polygon	51	2528
	**Water bodies**				
		Pond	Polygon	48	231
		Irrigation channels, distributaries, or drainage system (km)	Line	33	135.6
		Wells	Points	42	322
	**Road (km)**				
		Road	Line	51	707.2
		Lane (public)	Line	51	473.9
		Lanes (private)	Line	51	82.3
	Railroad		Line	2	3.7
**Social**					
	**Public places**				
		Chaupal	Point and Polygon	47	319
		Community center	Point and Polygon	18	21
	**Religious places**				
		Temple	Point and Polygon	40	248
		Mosque or Eidgah	Point and Polygon	16	94
		Madrassa	Point and Polygon	8	12
	**Others**				
		Old age home	Point and Polygon	13	13
		Monuments or landmark	Point	10	15
**Service**					
	Anganwadi child and mother care center		Point and Polygon	49	198
	Rural bank or mini bank or automated teller machine booth		Point and Polygon	11	17
	Kabristan or Shamshaanghat or cremation ground		Point and Polygon	42	64
	**Health facilities**				
		Community health center	Point and Polygon	1	1
		Primary health center	Point and Polygon	2	2
		Subcenter	Point and Polygon	18	18
		SOMAARTH clinics	Point and Polygon	5	5
		Veterinary clinic	Point and Polygon	16	16
		Public dispensary or Ayurvedic clinic	Point and Polygon	4	4
	**Educational institute**				
		School	Point and Polygon	49	172
		College	Point and Polygon	5	8
	**Others**				
		Water boosting station	Point and Polygon	36	48
		Village revenue office	Point and Polygon	9	9
		Bus depot or stand	Point and Polygon	3	3
		Railway station	Point and Polygon	2	2
		Petrol pump	Point and Polygon	10	15
		Post office	Point and Polygon	7	7
		Police station	Point and Polygon	2	2

^a^GIS: geographic information system.

**Table 2:** Concentration of habitations: villagewise distribution of the Nearest Neighbor Index.

Settlement typology	Nearest Neighbor Index	Village distribution, n (%)
Highly clustered	0-0.5	35 (68.6)
Clustered	0.6-0.9	15 (29.4)
Random	1.0	0
Regular	>1.0	1 (2.0)

On average, each village had 12 ([SD 9]; median 8; range 2-41) permanent litter areas and 75 wastewater stagnation points ([SD 39]; median 61; range 26-143 per village), and open defecation sites were marked in 43 villages. Food environment mapping carried out in 9 villages recorded 382 food stores with an average of 42 ([SD 40]; median 27; range 8-133) food stores per village.

Preliminary analysis of the settlement pattern using Nearest Neighbor Index (NNI) [34] indicated a clustered pattern (NNI<1.0) in 98% (50/51) villages; of them, 35 villages had highly concentrated settlements (NNI<0.5), and only 1 small village (Bazara Nagla) had an NNI of >1.0 (Table 2 and Multimedia Appendix 3). The cumulative area of the structural concentration of 51 villages was 2127 hectares (2127/23,788.7, 8.94%), encompassing 80.79% (37,979/47,007) of the total constructed land parcels (Multimedia Appendix 3).

### Space and Time Monitoring

The updated information on fine administrative boundaries (village, hamlets, land parcels) of the study area was missing from administrative records [35]. Official census maps (1:2 km scale) of the SOMAARTH area showed the boundaries of only 43 revenue villages; the district planning map helped delineate 4 additional small villages. The participatory GIS mapping process helped in the identification of 4 more small habitations (locally known as *nagla*) to make a total of 51 villages in the SOMAARTH DDESS. The absence of a formal subdivision of villages was a hindrance to the data collection process as the shape and size of the villages were organic and without any land use system.

Using geospatial tools, villages within the SOMAARTH site were subdivided into 760 sectors (range 5-26 sectors per village), with the area varying between 0.03 and 791.5 hectares. In a setting where no postal code system was in place, UIDs were created based on the georeferenced land parcels. Each enumerated land parcel was allotted a 19-digit-long UID covering the country, state, district, administrative block, village, sector, and land parcel number. Individuals were nested within the land parcel and given a computer-generated random 9-digit UID. Land parcel IDs had fixed geographies, whereas individual UIDs were kept independent to locations. All these were done with the objective of establishing a space, individual, and time monitoring system within the surveillance platform.

The social (caste categories such as schedule castes or tribes, backward communities, and general category) and economic (rich, middle, and poor classes) profile (socioeconomic profile) of all 676 sectors having households was assessed. Depending on the overall prevalence of socioeconomic classes in the SOMAARTH DDESS, if a sector had 1.5 times the average prevalence of a particular social or economic class, the sector was labeled as a dominant sector. Of all sectors, 61.8% (418/676) had a dominant caste and 34.8% (235/676) had a dominant economic class, with heterogeneity observed within and across the villages. Social class (ie, caste) was the major determinant of sector composition.

### Ground-Truthing–Based Data Verification and Refinement

Ground-truthing helped in the identification of both systematic and random errors in spatial and nonspatial data. Ground-truthing revealed that the data had positional and attribute errors, inconsistencies in land parcel boundary delineation, and lack of documentation of the vacant parcels. These errors had further escalated due to the temporal changes that occurred over 2 years between participatory mapping and preliminary verification exercise starting from 2011 to 2013. Out of the site-wide total land parcels, 23.53% land parcels (11,064/47,007) had size-related, 11.64% (5474/47,007) had shape-related, and 11.14% (5237/47,007) had location-related inaccuracies. In addition, 7990 vacant land parcels were left undocumented during the initial data collection exercise. In 12.09% (5687/47,007) of the land parcels, temporal changes like new construction (4640/5687, 81.6%) had occurred, with over three-fourth of these changes occurring during previous 6-24 months. The geospatial data of 1263 km of roads, including lanes and community pathways, was classified as per road typology, surface, and quality during the field visits. The final verification round conducted after the refinement of the data indicated that 4.90% (2303/47,007) of the land parcels still had positional inaccuracies due to the errors in size (141/47,007, 0.29%), shape (47/47,007, 0.09%), and location (2115/ 47,007, 4.50%) of the land parcels; 57.70% (1329/ 2303) of these errors recurred due to incorrect demarcation of individual property boundaries. Another 0.49% (235/47,007) of the land parcels were detected to have errors in attributes, and 184 more vacant land parcels were identified in this round.

### Physical Exposure to Harmful Environment

Physical exposure to harmful environment was assessed using two indicators calculated based on the geospatial mapping of the solid waste mounds and liquid waste spots in all of the 676 sectors having 32,631 households falling under the SOMAARTH DDESS area. The density of solid waste mounds and stagnant liquid waste puddles (both ≥1 m in diameter) within the sectors was calculated per 100 residents (median 2.7; 95% CI 4.2-5.4; range 0-60.9; Multimedia Appendix 3), and the Euclidian distance of households from the nearest solid waste dump or liquid waste puddle (in meters) was calculated for each of the households (median 29.4 m; 95% CI 64.2-67.9; range 1.5 m-2830.8 m). Village sectors were categorized as per the dominant socioeconomic classes of people living within them (proportion of a particular category more than 1.5 times the SOMAARTH average). The waste density and proximity variables calculated through GIS analysis were integrated with the socioeconomic data. The resultant analysis helped in characterizing the household-level condition of environmental sanitation vis-a-vis socioeconomic profile of the sectors. Table 3 presents the associations of sector-wide dominant social (caste) and economic classes with the harmful environmental indicators. Harmful environmental indicators such as higher sector waste density and household proximity (closeness) were significantly associated (*P*=.001) with the sector-wide dominant caste class. Waste spots were located at maximum distance from the plots or households in sectors inhabited by rich households.

**Figure 4:**
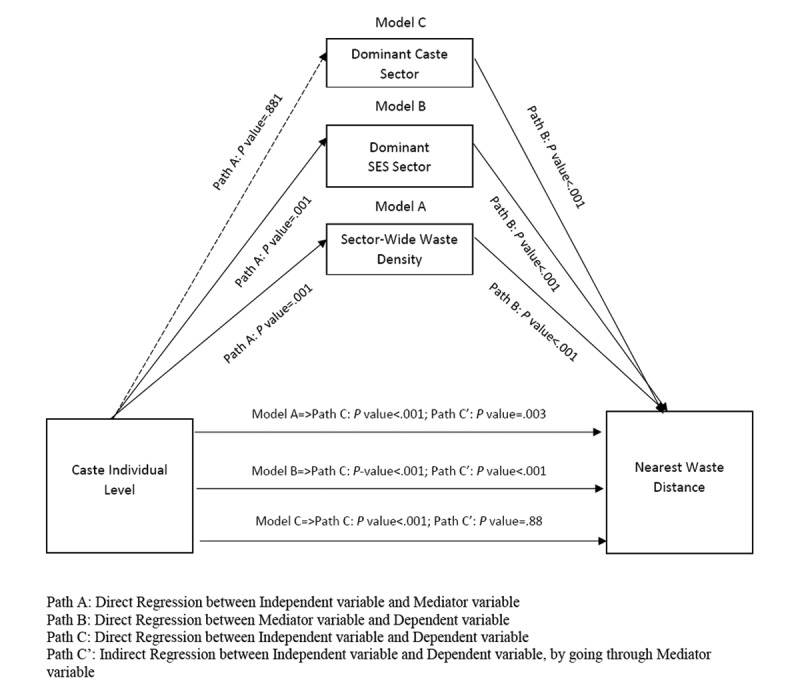
Structural equation model: mediational association between the caste of the household and sector-level environmental indicator, as well as social and economic dominance. SES: socioeconomic status.

Proximity (closeness) of the households to waste spots was examined using structural equation modeling [36] to explicitly describe the direct and indirect roles of various social and environmental determinants. The SES of the household was not found to be related to household proximity to waste spots either directly or indirectly after modeling for SES- and caste-dominant sectors and density of waste spots in the sector while adjusting for various household behavioral factors (household liquid and solid waste disposal practices, presence of a toilet, and source of drinking water within the households). However, the caste of the household was significantly associated with proximity to waste spots (*P*<.001). This effect was mediated through the SES dominance and waste density of the sector when adjusted for the previously mentioned household behavioral covariates (Figure 4). However, no significant association was found between household SES and proximity to waste spots (Table 4).

As part of another ongoing study [33], the nutrition (thinness and stunting) of a cohort of 612 children in the age group of 6-12 years was associated with the proximity of waste spots to the household, and the effects were mediated through caste dominance of the sector and religion of the household. The mediational effect was observed after adjusting for biologic factors like maternal height and sibship of the index child (Personal communication, Neha Gupta et al 2018—under publication).

**Table 3:** Relationship between socioeconomic class-dominated sectors (population subgroups) and environmental sanitation indicators.

Dominant^a^ sector		Value, n (%)	Sector waste density^b^, median	Nearest waste distance from the household^c^, median (m)
**Caste**				
	General	182 (26.9)	2.42^d^	30.9^d^
	Other backward castes	236 (34.9)	2.9^d^	29.8^d^
	Scheduled castes or scheduled tribes	110 (16.3)	2.6^d^	28.0^d^
**Socioeconomic status**				
	Rich	102 (15.1)	2.3	31.5^d^
	Middle	20 (3.0)	2.9	27.4^d^
	Poor	113 (16.7)	2.8	30.0^d^

^a^Dominant caste and socioeconomic status: a sector having 1.5 times the average prevalence of a particular economic or social class of the whole SOMAARTH Demographic, Development, and Environmental Surveillance Site.

^b^Sector waste density: number of solid waste mounds and stagnant liquid waste puddles (both >1 m in diameter) per 100 residents of a sector.

^c^Nearest waste distance from the household location (meters): distance of the nearest solid waste dump or water puddle (both >1 m in diameter), whichever was nearer.

^d^Significant at *P*=.001 (Kruskal-Wallis test).

**Table 4:** Structural equation model: mediational association between the caste of the household and sector-level environmental indicator, as well as social and economic dominance.

Covariates	Mediator variables, *P* value		
	Sector-level waste density (Model A)	Dominant SES^a^ sector (Model B)	Dominant caste sector (Model C)
Source of drinking water	Not significant	<.001	Not significant
Availability of toilet	<.001	<.001	.001
Liquid waste disposal	Not significant	Not significant	Not significant
Solid waste disposal	<.001	Not significant	.001
SES class	Not significant	Not significant	Not significant

^a^SES: socioeconomic status.

### Data Construction Cost

The total cost incurred in building SOMAARTH DDESS over the span of 7 years (2009-2015) was US $810,809 (12.6% spent on building the GIS infrastructure, including baseline data; 56.8% on census data construction; 8.2% on environmental monitoring; 4.6% on developing SOMAARTH software for census data storage; 5.2% on office essentials, including travel; 3.5% on other logistics or communication; and 10% on office utilities). The total cost of constructing the geospatial infrastructure, including baseline datasets, was US $102,666 (46% spent on technical staff salary, 26% on field worker salary, 7% on purchase of satellite imagery and GIS software, and 20% on office infrastructure and travel costs).

## Discussion

### Principal Findings

The unique features of the SOMAARTH DDESS are its architecture and capability to capture, store, and harmonize comprehensive datasets pertaining to the built environment, land use, access, weather and air quality, food environment, education, water and sanitation (liquid and solid waste), and health care services (public and private) for studying the individual-, household-, and community-level exposures and outcomes. Baiden [7] stated that the available surveillance platforms in developing countries such as MATLAB (Bangladesh), Filabavi (Vietnam), and Rakai (Uganda) are mostly the extension of surveillance systems for specific interventions. Similarly, the available literature on the methodology for the development of geospatial datasets reflects only the development of base maps for a particular intervention [17,18,37,38]. In contrast, we have described the methodology and architecture for building a GIS-integrated, comprehensive surveillance platform that can handle diverse health, developmental, and environmental issues in a convergent manner.

The overall approach and construct of the geospatial-enabled surveillance was feasible due to collective inputs from the interdisciplinary and transdisciplinary teams. Several authors have recently called for greater collaboration between disciplines to enrich research and explain the interaction and dynamics of environment, health, and well-being of individuals and societies, particularly in low- and middle-income countries [10,13,19]. Mixed methods involving participatory mapping, satellite imagery, and quantitative survey were adopted for capturing the accurate context and detailed composition of the study area. Participatory mapping can be achieved through several methods [28,37,38]. In our case, participatory mapping was achieved by utilizing the base maps (framework map) for overcoming the limitations of asymmetry (cartographic inaccuracies) and lack of reusability arising from hands-on mapping [28,30]. High-resolution satellite data along with community inputs helped in the identification of 4 new villages that were not present in official administrative records. Government records pertaining to fine administrative boundaries (village, hamlets, land parcels) are not regularly updated [19,35] and provide only aggregated data for revenue villages in developing countries. The community involvement provided insight into the local knowledge system, cultural practices, traditions, and customs [28], which were reflected in the organization of habitations and adjoining physical environment, identification of marginalized unnotified population groups, and access to traditional and cultural resources as well as community nomenclature, for example, *chaupal* (public places), *johad* (pond), *kos minar* (historical landmark), and *phirni* (ring road around the village).

Geospatial features were manually extracted from the pan-sharpened, high-resolution Quick bird satellite imagery. Makanga’s [38] research showed that manual digitization is the most effective and a cheaper way for health GIS data constructions at low-resource settings. For simplifying the task of mapping in morphologically complex villages, we adopted principles of spatial generalization [39] for the delineation of land parcels and village boundaries. Unlike the urban areas, the process of geocoding could not be applied in most of the rural areas of developing countries as postal codes for properties were not available [40].

The systematic methodology adopted for subdividing the villages into sectors on the lines of urban areas helped in building a system for georeferenced UIDs as well as in identifying socially homogenous community clusters (418/676, 61.8%, sectors) within the villages (Table 3). The computed physical exposure to harmful environment (proximity to waste spots) was significantly associated with the caste of the household, a social class indicator within the villages; the effect was mediated through SES dominance and waste spot density of the sector. Household behavioral factors like the source of water, presence of a toilet, and waste disposal practices were directly affecting these relationships. (see Table 4). These environmental factors, in turn, had the potential to influence the health and nutrition of the household members [41]. The INCLEN SOMAARTH surveillance platform was being used to prospectively assess the health outcomes of the national flagship intervention program “Clean-India (Swatch Bharat)” [42]. Projects implemented at the SOMAARTH DDESS have the potential to harness granular data related to diverse aspects of demography, development, and environment. Multimedia Appendix 4 shows coarse-resolution administrative maps of the surveillance villages that were the only spatial data available with the government. The top left and right maps are fine-resolution sector map and built-up area map, respectively, of surveillance villages that helped characterize land use; the middle one shows liquid and solid waste spots mapping that was overlaid on the land use map, and the bottom one depicts the location of food stores and their distance from the water-stagnant areas.

The community- and household-level exposure details could, therefore, be used to explain and quantify diverse societal determinants of health; profiling of sociocultural and economic status of sectors within villages also opened up opportunities for designing and implementation of complex intervention studies incorporating social determinants [14-16].

Several studies have highlighted the possibilities of generating erroneous geospatial data and exposure misclassification due to the nonavailability of valid and quality administrative data and the absence of thorough ground-truthing exercises [43-45]. The settlement pattern was highly compact across the site (see Table 2); 80.79% (37,979/47,007) of the total built structures were concentrated in 8.94% (2127 hectare/23788.7 hectare) of the total area, which was consistent with previous observations from developing countries [46]. Also, the empirical datasets reflected rapid expansion of built-up area in adjoining agricultural belts. Ground-truthing exercises were, therefore, kept as an integral step in the methodology for addressing the potential positional, attribute (location, size, and shape), and temporal discrepancies [43,44]. The first round of geospatial data verification revealed that 88.50 % (41,601/47,007) of the total land parcels were accurately marked for their location, but only 64.5% (30,320/47,007) of the land parcels were correctly mapped in terms of their relative size and shape. We faced the additional challenge of nonavailability of physical demarcation in almost one-third (27.0%, 12,692/47,007) of the land parcels. The physical demarcation of land parcels affected image interpretation and, thus, the quality of land parcel data. Ground-truthing of the land parcel data in rural settings of countries like India shall, therefore, remain an essential step for finalizing spatial datasets [43]. However, as reported earlier, the use of satellite imagery resulted in high degrees (>95%) of positional accuracies for features such as water bodies and roads [45].

A licensed proprietary software Arc GIS 10.3 (ESRI, Redland, CA, USA) along with the open source (QGIS) software was used to expedite the digitization of large volumes of geospatial data without adding burden on limited financial resources. Similar strategy for cost minimization was also tried at other resource-constrained settings [47]. Although we could not perform any direct comparison for cost incurred in developing similar surveillance platforms in other low- and middle-income countries, investments in SOMAARTH-like comprehensive platforms are likely to be far more useful than establishing categorical surveillance systems criticized for their limited capacities and sustainability [2].

We identified three major challenges in building fine-resolution geospatial datasets for a surveillance system in a scientific manner in resource-constrained settings. First, administrative health datasets were not available, and varied spatial data frames were followed at different data sources; therefore, the high-resolution vectors prepared through satellite imagery could not be properly integrated with the administrative datasets. Due to such problems, the recent report from National Institute of Advanced Studies [48] has advocated the initiation of a unified spatial framework under National GIS in the country; geocoding is not possible in these areas due to the lack of an address system in rural areas. Second, due to the compact nature of the settlement in our villages, we faced difficulty in using Global Positioning System (GPS) [17,21]. In the absence of GPS coordinates, characterization of satellite imagery was a challenging task. Third, there was a shortage of skilled personnel for long-term work engagements in rural areas [19,20]. Although a period of 5 years was required to set up SOMAARTH DDESS, we believe that based on the learnings, subsequent endeavors can be accomplished in much shorter periods using Web GIS and advanced GPS recorders.

### Way Forward

The granular data generated through the SOMAARTH surveillance platform could be harnessed in designing complex research studies taking into account social determinants of diseases and health; furthermore, environmental and behavioral interventions could be targeted at subvillage and household levels [49-52], which are presently constrained due to data unavailability. The land use datasets could also be harmonized with the available international GIS-integrated surveillance sites [53] to promote multicentric spatial epidemiological studies.

## Appendix

Multimedia Appendix 1 SOMAARTH website—www.sommarth.org.

Multimedia Appendix 2 Architecture of the SOMAARTH Demographic, Development, and Environmental Surveillance Site.

Multimedia Appendix 3 Supplementary file; profile of villages at SOMAARTH DDESS, Palwal, India.

Multimedia Appendix 4 Detailed context and composition stored in the SOMAARTH Demographic, Development, and Environmental Surveillance Site geospatial data layers.

## Abbreviations

DDESS: Demographic, Development, and Environmental Surveillance Site
GIS: geographic information system
GPS: Global Positioning System
INCLEN: The International Clinical Epidemiology Network
NNI: Nearest Neighbor Index
PM _2.5_: particulate matter 2.5
SES: socioeconomic status
UID: unique identification
