# Supplementary figures and images for: Establishing a Demographic, Development and Environmental Geospatial Surveillance Platform in India: Planning and Implementation
Source: JMIR Public Health Surveill. 2018 Oct 5;4(4):e66. doi: 10.2196/publichealth.9749 (PMC6231830; doi:10.2196/publichealth.9749)

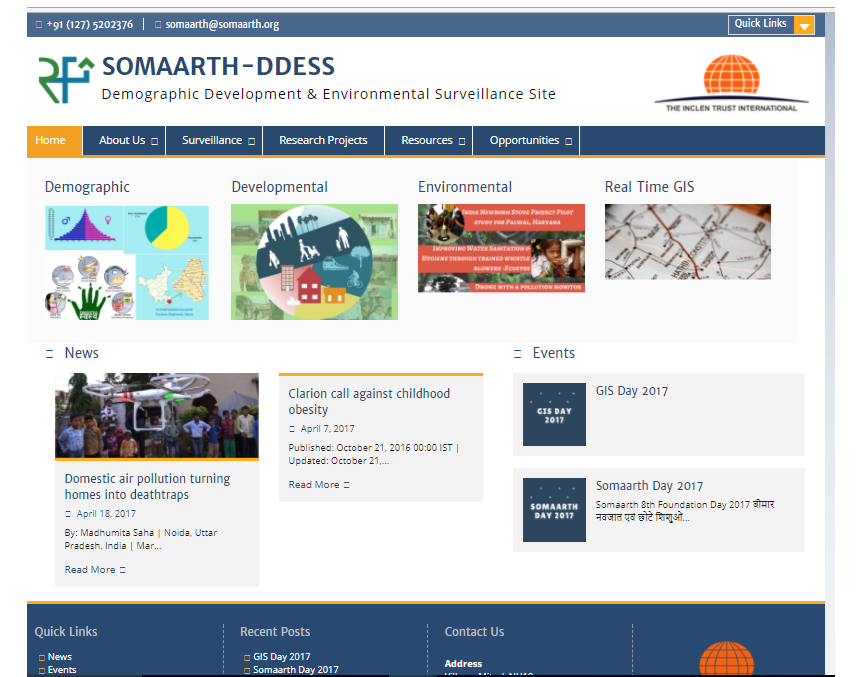

Supplement: Multimedia Appendix 1 [file publichealth_v4i4e66_app1.png]

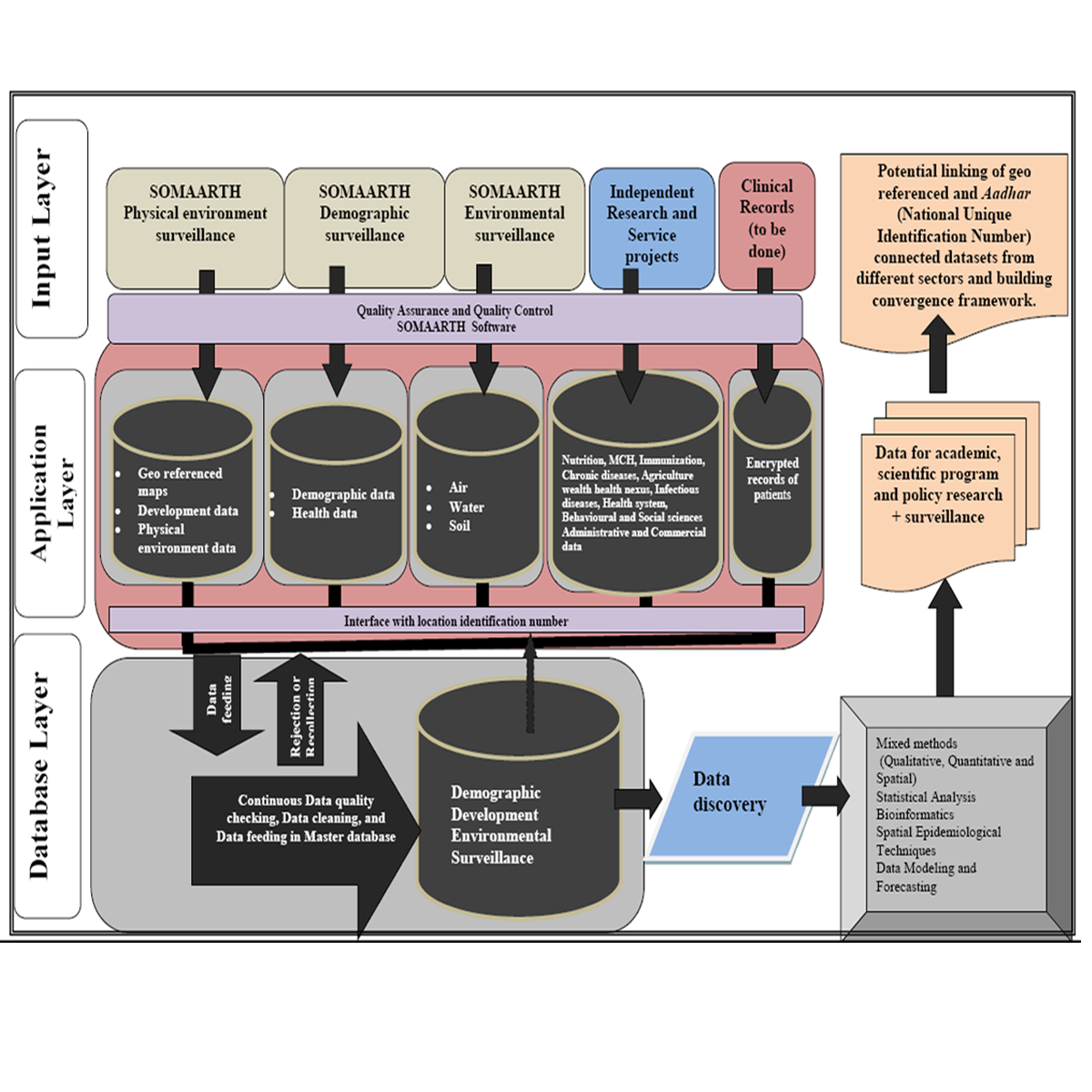

Supplement: Multimedia Appendix 2 [file publichealth_v4i4e66_app2.png]

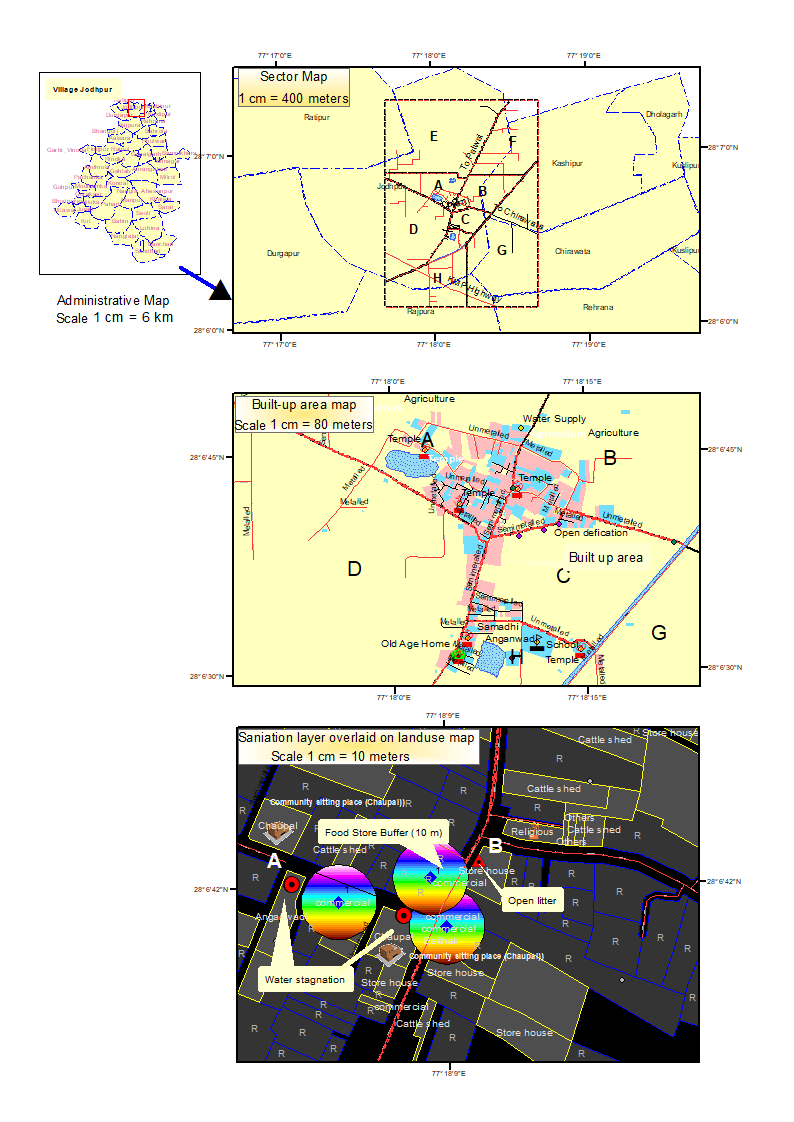

Supplement: Multimedia Appendix 4 [file publichealth_v4i4e66_app4.png]
