# Supplementary material for: Establishing a Demographic, Development and Environmental Geospatial Surveillance Platform in India: Planning and Implementation
Source: JMIR Public Health Surveill. 2018 Oct 5;4(4):e66. doi: 10.2196/publichealth.9749 (PMC6231830; doi:10.2196/publichealth.9749)
Supplement: Multimedia Appendix 3 [file publichealth_v4i4e66_app3.pdf]

| Table S1: Village wise distribution of population, land parcel, subunits and household population occupancy | | | | | | | | | |
|---|---|---|---|---|---|---|---|---|---|
| Village | Population | Land parcel | | | | Subunit features[a] | | | Household population occupancy |
| | | Total | Residential | Non residential | Mixed | Total | Residential | Non residential | |
| Andhrola | 3545 | 638 | 392 (61.4) | 218 (34.2) | 28 (4.9) | 819 | 566 (69.1) | 253 (30.9) | 6 |
| Aurangabad | 10,281 | 3202 | 1573 (49.1) | 1489 (46.5) | 140 (4.4) | 3690 | 1976 (53.6) | 1714 (46.4) | 5 |
| Bahin | 7484 | 1951 | 1057 (54.2) | 832 (42.6) | 62 (3.2) | 2260 | 1353 (59.9) | 907 (40.1) | 6 |
| Bajaranangla | 89 | 25 | 13 (52.0) | 11(44.0) | 1 (4.0) | 29 | 17 (58.6) | 12 (41.4) | 5 |
| Bajdapahari | 627 | 183 | 83(45.4) | 83 (45.4) | 17 (9.3) | 222 | 115 (51.8) | 107 (48.2) | 5 |
| Bamnikhera | 9992 | 2747 | 1488 (54.2) | 1071 (39.0) | 188 (6.8) | 3439 | 2044 (59.4) | 1395 (40.6) | 5 |
| Banchari | 11,288 | 3068 | 1734 (56.5) | 1174 (38.3) | 160 (5.2) | 3538 | 2136 (60.4) | 1402 (39.6) | 5 |
| Behrola | 2386 | 551 | 295 (53.5) | 221 (40.1) | 35 (6.4) | 689 | 426 (61.8) | 263 (38.2) | 6 |
| Bhamarikhera | 287 | 54 | 28 (51.9) | 19 (35.2) | 7 (12.9) | 70 | 42 (60.0) | 28 (40.0) | 7 |
| Bhamrolajogi | 1280 | 341 | 177 (51.9) | 144 (42.2) | 20 (5.9) | 394 | 223 (56.6) | 171 (43.4) | 6 |
| Bhanguri | 1473 | 455 | 203 (44.6) | 232 (51.0) | 20 (4.4) | 525 | 259 (49.3) | 266 (50.6) | 6 |
| Bhudpur | 1080 | 206 | 135(65.5) | 63 (30.6) | 8 (3.9) | 269 | 194 (72.1) | 75 (27.9) | 6 |
| Chirawata | 1647 | 439 | 240 (54.7) | 187 (42.6) | 12 (2.7) | 507 | 297 (58.6) | 210 (41.4) | 6 |
| Dholagarh | 1471 | 333 | 222 (66.7) | 94 (28.2) | 17 (5.1) | 410 | 289 (70.5) | 121 (29.5) | 5 |
| Durgapur | 2438 | 618 | 364 (58.9) | 224 (36.2) | 30 (4.9) | 767 | 492 (64.1) | 275 (35.9) | 5 |
| Firozpurrajput | 1700 | 418 | 224 (53.6) | 168 (41.0) | 26 (6.2) | 504 | 305 (60.5) | 199 (39.5) | 6 |
| Garhivinoda | 618 | 184 | 78 (42.4) | 99 (53.8) | 7 (3.8) | 206 | 94 (45.6) | 112 (54.4) | 7 |
| Gehlab | 6532 | 1800 | 929 (51.6) | 783 (43.5) | 88 (4.9) | 2074 | 1177 (56.8) | 897 (43.2) | 6 |
| Gohpur | 2603 | 496 | 243 (49.0) | 182 (36.7) | 71 (14.3) | 710 | 421 (59.3) | 289 (40.7) | 6 |
| Gopalgarh | 927 | 255 | 118 (46.3) | 125 (49.0) | 12 (4.7) | 325 | 178 (54.8) | 147 (45.2) | 5 |
| Guraksar | 5780 | 1075 | 678 (63.0) | 324 (30.1) | 73 (6.8) | 1433 | 1003 (70.0) | 430 (30.0) | 6 |
| Jodhpur | 1704 | 433 | 227 (52.4) | 193 (44.6) | 13 (3.0) | 502 | 289 (57.6) | 213 (42.4) | 6 |
| Jurali | 2132 | 419 | 262 (62.5) | 141 (33.7) | 16 (3.8) | 515 | 350 (68.0) | 165 (32.0) | 6 |
| Kalsada | 2386 | 587 | 348 (59.9) | 204 (34.8) | 35 (6.96) | 683 | 435 (63.7) | 248 (36.3) | 5 |
| Kashipur | 735 | 207 | 108(52.2) | 92 (44.4) | 7 (3.38) | 237 | 137 (57.8) | 100 (42.2) | 5 |
| Khatela | 1607 | 383 | 218 (56.9) | 155 (40.5) | 10 (2.6) | 449 | 280 (62.4) | 169 (37.6) | 6 |
| Khilluka | 3892 | 721 | 503 (69.7) | 181 (25.1) | 37 (5.13) | 902 | 679 (75.3) | 223 (24.7) | 6 |
| Kondal | 6103 | 1740 | 911 (52.4) | 784 (45.1) | 45 (2.58) | 1947 | 1113 (57.2) | 834 (42.8) | 5 |
| Kot | 9709 | 1618 | 1113 (68.8) | 432 (26.7) | 73 (4.5) | 2034 | 1490 (73.3) | 544 (26.7) | 7 |
| Kushlipur | 3727 | 1112 | 524 (47.1) | 534 (48.0) | 54 (4.85) | 1300 | 677 (52.1) | 623 (47.9) | 6 |
| Lohina | 2967 | 840 | 519 (61.7) | 306 (36.4) | 15 (1.78) | 926 | 603 (65.1) | 323 (34.9) | 5 |
| Manpur | 8934 | 2214 | 1343 (60.7) | 803 (36.3) | 68 (3.07) | 2516 | 1624 (64.5) | 892 (35.5) | 6 |
| Mithaka | 990 | 159 | 92(57.9) | 54 (34.0) | 13 (8.17) | 218 | 146 (67.97) | 72 (33.0) | 7 |
| Mitrol | 4980 | 1364 | 773 (56.7) | 501 (36.7) | 90 (6.0) | 1629 | 996 (61.14) | 633 (38.9) | 5 |
| Mohadamka | 1631 | 253 | 131 (51.8) | 77 (30.4) | 45 (17.8) | 396 | 260 (65.65) | 136 (34.3) | 6 |

| | | | | | | | | | |
|---|---|---|---|---|---|---|---|---|---|
| Nangaljat | 4781 | 1178 | 736 (62.4) | 392 (33.3) | 50 (4.2) | 1379 | 915 (66.35) | 464 (33.6) | 5 |
| Nanglaahsanpur | 2817 | 543 | 361 (66.5) | 165 (30.4) | 17 (3.1) | 652 | 465 (71.3) | 187 (28.7) | 6 |
| Pachanka | 3312 | 700 | 337 (48.1) | 291 (41.6) | 72 (10.3) | 901 | 508 (56.38) | 393 (43.6) | 7 |
| Pahari | 2055 | 549 | 313(57.0) | 222(40.4) | 14 (2.6) | 609 | 373 (61.2) | 236 (38.8) | 6 |
| Phulwari | 5810 | 1309 | 539 (41.1) | 546 (41.7) | 224 (17.1) | 1901 | 983 (51.7) | 918 (48.3) | 6 |
| Rajpura | 3411 | 616 | 372 (60.9) | 207 (33.6) | 37 (6.0) | 848 | 577 (68.0) | 271 (32.0) | 6 |
| Ratipur | 1950 | 510 | 270 (52.9) | 218 (42.7) | 22 (4.3) | 638 | 363 (56.9) | 275 (43.1) | 5 |
| Rehrana | 2937 | 697 | 369 (52.9) | 285 (40.9) | 43 (6.2) | 855 | 495 (57.9) | 360 (42.1) | 6 |
| Rindika | 1510 | 359 | 188 (52.4 | 146 (40.7) | 25 (7.0) | 457 | 275 (60.2) | 182 (39.8) | 5 |
| Sarai | 4720 | 789 | 529 (67.0) | 216 (27.4) | 44 (5.6) | 1038 | 752 (72.4) | 286 (27.6) | 6 |
| Seoli | 4508 | 1057 | 623 (58.9) | 400 (37.8) | 34 (3.2) | 1251 | 804 (64.3) | 447 (35.7) | 6 |
| Sondhad | 11,402 | 2512 | 1626 (64.7) | 814 (32.4) | 72 (2.9) | 3162 | 2244 (71.0) | 918 (29.0) | 5 |
| Srinagar | 436 | 237 | 110 (46.4) | 124 (52.3) | 3 (1.3) | 248 | 121 (48.8) | 127 (51.2) | 4 |
| Sunder nagar | 419 | 97 | 66(68.0) | 28 (28.9) | 3 (3.1) | 115 | 84 (73.0) | 31 (26.9) | 5 |
| Tikribrahmin | 6360 | 1486 | 817(55.0) | 607 (40.8) | 62 (4.2) | 1794 | 1089 (60.7) | 705 (39.3) | 6 |
| Uttawar | 18,249 | 3279 | 1761 (53.7) | 1255 (38.3) | 263 (8.0) | 4383 | 2755 (62.9) | 1628 (37.1) | 7 |
| **Total** | **1,99,702** | **47,007** | **26,363 (56.1)** | **18,116 (38.5)** | **2528 (5.4)** | **57,365** | **35,489 (619)** | **21,876 (38.1)** | **6** |
| **Mean** | **3916** | **922** | **517** | **355** | **50** | **1125** | **696** | **429** | **6** |
| **SD** | **3673** | **858** | **479** | **345** | **56** | **1045** | **644** | **416** | **0.7** |
| **Median** | **2603** | **587** | **348** | **218** | **34** | **710** | **465** | **266** | **6** |
| **Range (min)** | **89** | **25** | **13** | **11** | **1** | **29** | **17** | **12** | **4** |
| **Range (max)** | **18,249** | **3279** | **1761** | **1489** | **263** | **4383** | **2755** | **1714** | **7** |

**[a]Figure include 5441 (4491 locked and 950 abandoned) unused structures at the time of survey.**

Table S3: Village wise characterization and profiling of structural concentration zone

| Sl No | Village Name | Village area [b] (ha) | Structural Concentration Zone[a] (SCZ) Buffer (m) | Area covered ha (% of total village area) | Number of land Parcel (% of total) | Land parcel density (lp/ha) |
|---|---|---|---|---|---|---|
| 1 | Andhrola | 287.5 | 300 | 28 (9.8) | 537 (84.2) | 19 |
| 2 | Aurangabad | 1630.2 | 650 | 133 (8.1) | 2614 (81.6) | 20 |
| 3 | Bahin | 2050.4 | 550 | 95 (4.6) | 1646 (84.4) | 17 |
| 4 | Bajaranangla | 23.3 | 200 | 13 (53.9) | 20 (80.0) | 2 |
| 5 | Bajdapahari | 263.3 | 300 | 20 (7.5) | 149 (81.4) | 8 |
| 6 | Bamnikhera | 764.1 | 600 | 113 (14.8) | 2206 (80.3) | 20 |
| 7 | Banchari | 1514.8 | 500 | 79 (5.2) | 2516 (82.0) | 32 |
| 8 | Behrola | 283.7 | 550 | 113 (39.9) | 445(80.8) | 4 |
| 9 | Bhamarikhera | 31.1 | 200 | 13 (40.4) | 46 (85.0) | 4 |
| 10 | Bhamrolajogi | 300.5 | 175 | 10 (3.2) | 266 (78.0) | 28 |
| 11 | Bhanguri | 400.5 | 220 | 15 (3.8) | 378 (83.1) | 25 |
| 12 | Bhudpur | 108.3 | 125 | 5 (4.5) | 157 (76.2) | 32 |
| 13 | Chirawata | 124.9 | 200 | 13 (10.1) | 362 (82.5) | 29 |
| 14 | Dholagarh | 41.9 | 300 | 28 (67.5) | 268 (80.5) | 9 |
| 15 | Durgapur | 340.3 | 250 | 20 (5.8) | 473 (76.5) | 24 |
| 16 | Firozpur rajput | 300 | 200 | 13(4.2) | 355 (84.9) | 28 |
| 17 | Garhivinoda | 191.5 | 250 | 20 (10.3) | 144 (78.3) | 7 |
| 18 | Gehlab | 1045.2 | 400 | 50 (4.8) | 1484 (82.4) | 30 |
| 19 | Gohpur | 112.2 | 250 | 20 (17.5) | 418 (84.3) | 21 |
| 20 | Gopalgarh | 270.1 | 350 | 38 (14.2) | 194 (76.1) | 5 |
| 21 | Guraksar | 354.5 | 350 | 38 (10.9) | 895 (83.3) | 23 |
| 22 | Jodhpur | 277 | 300 | 28 (10.2) | 339(78.3) | 12 |
| 23 | Jurali | 102.5 | 200 | 13 (12.3) | 325 (77.6) | 26 |
| 24 | Kalsada | 294.2 | 250 | 20 (6.7) | 492 (83.8) | 25 |
| 25 | Kashipur | 87.9 | 150 | 7 (8.0) | 165 (79.7) | 23 |
| 26 | Khatela | 274.9 | 225 | 16 (5.8) | 296 (77.3) | 19 |
| 27 | Khilluka | 181.9 | 250 | 20 (10.8) | 572(79.3) | 29 |
| 28 | Kondal | 1199.9 | 450 | 64 (5.3) | 1393 (80.1) | 22 |
| 29 | Kot | 949.1 | 500 | 79 (8.3) | 1311 (81.0) | 17 |
| 30 | Kushlipur | 333.3 | 500 | 79 (23.6) | 895 (80.5) | 11 |
| 31 | Lohina | 1337.8 | 500 | 79 (5.9) | 635 (75.6) | 8 |
| 32 | Manpur | 1577 | 450 | 64 (4.0) | 1814 (81.9) | 29 |
| 33 | Mithaka | 51.8 | 140 | 6 (11.9) | 126 (79.2) | 20 |
| 34 | Mitrol | 503.3 | 400 | 50(10.0) | 1108 (81.2) | 22 |
| 35 | Mohadamka | 94.6 | 180 | 10 (10.8) | 206 (81.4) | 20 |
| 36 | Nangaljat | 524 | 400 | 50 (9.6) | 963 (81.7) | 19 |
| [illegible] | [illegible] | [illegible] | [illegible] | [illegible] | [illegible] | [illegible] |

| ...ghbour index: ...ean [b] | NN Index[c] |
|---|---|
| | 0.48 |
| | 0.39 |
| | 0.30 |
| | 1.01 |
| | 0.51 |
| | 0.44 |
| | 0.86 |
| | 0.36 |
| | 0.50 |
| | 0.40 |
| | 0.44 |
| | 0.69 |
| | 0.51 |
| | 0.46 |
| | 0.42 |
| | 0.36 |
| | 0.39 |
| | 0.39 |
| | 0.62 |
| | 0.45 |
| | 0.49 |
| | 0.44 |
| | 0.63 |
| | 0.43 |
| | 0.53 |
| | 0.29 |
| | 0.56 |
| | 0.43 |
| | 0.48 |
| | 0.54 |
| | 0.39 |
| | 0.30 |
| | 0.74 |
| | 0.45 |

**Table S4: Village wise waste density**

| Block | Village | Solid waste (N) | Liquid waste (N) | Total waste (N) | Total Population (N) | Solid waste density per 100 population | Liquid waste density per 100 population | Total waste density per 100_population |
|---|---|---|---|---|---|---|---|---|
| Hathin | Andhrola | 17 | 69 | 86 | 3545 | 0.48 | 1.95 | 2.43 |
| Hathin | Bahin | 43 | 215 | 258 | 7484 | 0.57 | 2.87 | 3.45 |
| Hathin | Bazara Pahari | 12 | 14 | 26 | 627 | 1.91 | 2.23 | 4.15 |
| Hathin | Bhamrola Jogi | 20 | 102 | 122 | 1280 | 1.56 | 7.97 | 9.53 |
| Hathin | Bhanguri | 8 | 39 | 47 | 1473 | 0.54 | 2.65 | 3.19 |
| Hathin | Budhpur | 6 | 33 | 39 | 1080 | 0.56 | 3.06 | 3.61 |
| Hathin | Firojpur Rajput | 9 | 46 | 55 | 1700 | 0.53 | 2.71 | 3.24 |
| Hathin | Garhi Vinoda | 7 | 11 | 18 | 618 | 1.13 | 1.78 | 2.91 |
| Hathin | Gehlab | 39 | 196 | 235 | 6532 | 0.60 | 3.00 | 3.60 |
| Hathin | Gohpur | 19 | 91 | 110 | 2603 | 0.73 | 3.50 | 4.23 |
| Hathin | Gurakshar | 16 | 176 | 192 | 5780 | 0.28 | 3.04 | 3.32 |
| Hathin | Jurali | 9 | 57 | 66 | 2132 | 0.42 | 2.67 | 3.10 |
| Hathin | kalsara | 17 | 93 | 110 | 2386 | 0.71 | 3.90 | 4.61 |
| Hathin | Khilluka | 13 | 140 | 153 | 3892 | 0.33 | 3.60 | 3.93 |
| Hathin | Kondal | 27 | 256 | 283 | 6103 | 0.44 | 4.19 | 4.64 |
| Hathin | Kot | 44 | 157 | 201 | 9709 | 0.45 | 1.62 | 2.07 |
| Hathin | Manpur | 43 | 348 | 391 | 8934 | 0.48 | 3.90 | 4.38 |
| Hathin | Meethaka | 5 | 53 | 58 | 990 | 0.51 | 5.35 | 5.86 |
| Hathin | Modhamka | 4 | 45 | 49 | 1631 | 0.25 | 2.76 | 3.00 |
| Hathin | Nangalijat | 40 | 156 | 196 | 4781 | 0.84 | 3.26 | 4.10 |
| Hathin | pachanka | 21 | 95 | 116 | 3312 | 0.63 | 2.87 | 3.50 |
| Hathin | pahari | 21 | 84 | 105 | 2055 | 1.02 | 4.09 | 5.11 |
| Hathin | Rindka | 1 | 46 | 47 | 1510 | 0.07 | 3.05 | 3.11 |
| Hathin | Uttawar | 77 | 360 | 437 | 18249 | 0.42 | 1.97 | 2.39 |
| Hodal | Aurangabad | 74 | 293 | 367 | 10281 | 0.72 | 2.85 | 3.57 |
| Hodal | Bajara Nangla | 3 | 3 | 6 | 89 | 3.37 | 3.37 | 6.74 |
| Hodal | Bamnikhera | 60 | 238 | 298 | 9992 | 0.60 | 2.38 | 2.98 |
| Hodal | Banchari | 32 | 241 | 273 | 11288 | 0.28 | 2.14 | 2.42 |
| Hodal | Behrola | 16 | 64 | 80 | 2386 | 0.67 | 2.68 | 3.35 |
| Hodal | Bhamarikheda | 5 | 20 | 25 | 287 | 1.74 | 6.97 | 8.71 |
| Hodal | Gopalgarh | 7 | 10 | 17 | 927 | 0.76 | 1.08 | 1.83 |
| Hodal | Khatela | 17 | 1 | 18 | 1607 | 1.06 | 0.06 | 1.12 |
| Hodal | Lohina | 26 | 138 | 164 | 2967 | 0.88 | 4.65 | 5.53 |
| Hodal | Mitrol | 19 | 125 | 144 | 4980 | 0.38 | 2.51 | 2.89 |
| Hodal | Nangla Ahsanpur | 33 | 152 | 185 | 2817 | 1.17 | 5.40 | 6.57 |
| Hodal | Phulwari | 48 | 234 | 282 | 5810 | 0.83 | 4.03 | 4.85 |
| Hodal | sarai | 21 | 116 | 137 | 4720 | 0.44 | 2.46 | 2.90 |
| Hodal | Seoli | 39 | 103 | 142 | 4508 | 0.87 | 2.28 | 3.15 |
| Hodal | Sondh | 73 | 223 | 296 | 11402 | 0.64 | 1.96 | 2.60 |
| Hodal | Srinagar | 14 | 10 | 24 | 436 | 3.21 | 2.29 | 5.50 |
| Hodal | Sundarnagar | 5 | 9 | 14 | 419 | 1.19 | 2.15 | 3.34 |
| [illegible] | [illegible] | [illegible] | [illegible] | [illegible] | [illegible] | [illegible] | [illegible] | [illegible] |
